# Supplementary material for: Interaction between Nanoparticles, Membranes and Proteins: A Surface Plasmon Resonance Study
Source: Int J Mol Sci. 2022 Dec 29;24(1):591. doi: 10.3390/ijms24010591 (PMC9820549; doi:10.3390/ijms24010591)
Supplement: Supplementary file 1 [file ijms-24-00591-s001.zip › ijms-1900841-supplementary.pdf]

Article

Interaction between Gold Nanoparticles, Membranes and Proteins: A Surface Plasmon Resonance Study

Erenildo Ferreira de Macedo [1,2], Nivia Salles Santos [1,2], Lucca Silva Nascimento [1], Yanxia Hou-Broutin [2], Matheus Sacilotto de Moura [1], Alexandre Martins Isaias dos Santos [1], Dayane Batista Tada [1]

[1] Federal University of São Paulo (UNIFESP), Institute of science and Technology, São Paulo, Brazil, e.macedo@unifesp.br
[2] Affiliation 2; e-mail@e-mail.com
* Correspondence: e-mail@e-mail.com; Tel.: (optional; include country code; if there are multiple corresponding authors, add author initials)

Supporting information

1. Dissociation constant (k_{off})

The interaction between nanoparticles and different kind of proteins immobilized on the sensor surface was analyzed using the dissociation constant (k_{off}). First, the interactions between nanoparticles and proteins were observed through kinetic curves where the dissociation step was used to obtain the k_{off}.

The dissociation step on the kinetics curve (**figure 1**) was fitting using the equation 1. Where ***Rd*** is the response of the dissociation step, ***Ro*** is the exponencial gain and k_{off} is the dissociation constant.

$$Rd = Ro\ .\ exp^{-Koff\ .\ t} \qquad \textit{equation 1}$$

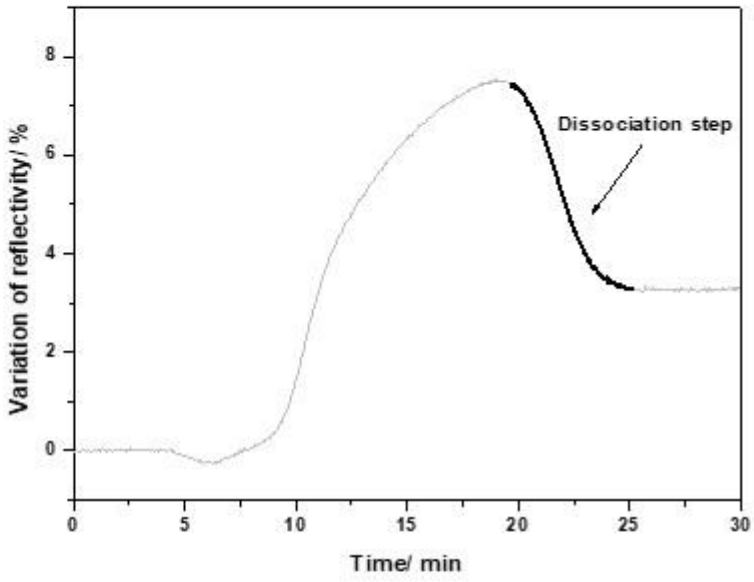

Figure S1: Kinetic curve of the interaction between nanoparticle and protein. Dissociation step (black).

2. Surface Concentration Density (*Γ*)

The interaction between successive injections of nanoparticles and different types of membrane was analyzed to obtain the surface concentration density (**Γ**).

The greater reflectivity variation corresponds to the highest concentration on the sensor surface, therefore, this value is used to obtain **Γ**.

Firstly, it was determinated the decay length ($\boldsymbol{l_d}$), equation 2.

$$l_d = \frac{\lambda/2\pi}{[-n^4{}_{eff}/(n^2{}_{eff} + \varepsilon_{metal})]^{1/2}} \qquad \textit{equation 2}$$

Where $\boldsymbol{n_{eff}}$ is the refractive index measured in the equipment, $\boldsymbol{\varepsilon_{metal}}$ is the characteristic parameter of the metal and **λ** is the wavelength.

The equation 3 describe the thickness of the sensor surface layer.

$$d = -\left(\frac{l_d}{2}\right) \ln\left(1 - \left[\frac{n_{eff} - n_b}{n_a - n_b}\right]\right) \qquad \textit{equation 3}$$

Where $\boldsymbol{d}$ is the thickness of the sensor surface layer, $\boldsymbol{n_b}$ is the refractive index of the solvent and $\boldsymbol{n_a}$ is the refractive index of the material used.

The equation 4 describe the surface concentration density (**Γ**).

$$\Gamma = d\left(\frac{n_a - n_b}{d_n/d_c}\right) \qquad \textit{equation 4}$$

Where $\boldsymbol{d_n/d_c}$ is the Increment refractive index of gold nanoparticles. This parameter is the slope obtained on the analitical curve. This analitical curve was obtained analyzing the refractive index of different concentrations of gold nanoparticles (figure 2)

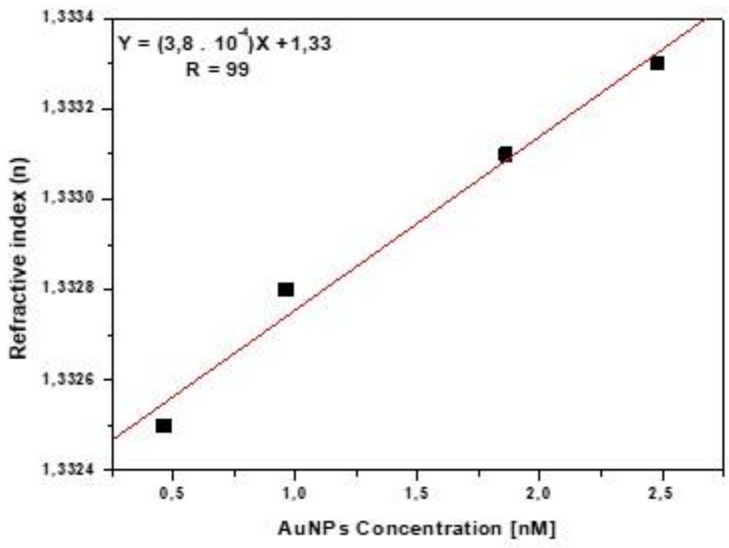

Figure S2: Measurement of refractive index at varied concentrations of gold NPs. The slope is the parameter dn/dc.
